# Supplementary material for: Investigation of Cation Exchange Behaviors of FAxMA1−xPbI3 Films Using Dynamic Spin-Coating
Source: Materials (Basel). 2021 Oct 26;14(21):6422. doi: 10.3390/ma14216422 (PMC8585367; doi:10.3390/ma14216422)
Supplement: Supplementary file 1 [file materials-14-06422-s001.zip › materials-1426131-supplementary.pdf]

Communication

Investigation of Cation Exchange Behaviors of $FA_xMA_{1-x}PbI_3$ Films Using Dynamic Spin-Coating

HyangMi Yu [1,†], Byeong-Geun Jeong [1,†], Dae-Young Park [2], Seong-Chu Lim [1,3,*], Gon Namkoong [1,4,*] and Mun-Seok Jeong [2,5,*]

1 Department of Energy Science, Sungkyunkwan University, Suwon 16419, Korea; gidal0072@skku.edu (H.M.Y.); zinzza228@skku.edu (B.G.J.)
2 Department of Physics, Hanyang University, Seoul 04763, Korea; parkdy004@hanyang.ac.kr
3 Department of Smart Fab. Technology, Sungkyunkwan University, Suwon 16419, Korea
4 Applied Research Centre, Department of Electrical and Computer Engineering, Old Dominion University, 12050 Jefferson Avenue, Newport News, VA 23693, USA
5 Department of Energy Engineering, Hanyang University, Seoul 04763, Korea
† H.M.Y and B.G.J contributed equally to this work.
* Correspondence: seonglim@skku.edu (S.C.L.); gnamkoon@odu.edu (G.N.); mjeong@hanyang.ac.kr (M.S.J.)

Citation: Yu, H.M.; Jeong, B.-G.; Park, D.-Y.; Lim, S.-C.; Namkoong, G.; Jeong, M.-S. Investigation of Cation Exchange Behaviors of $FA_xMA_{1-x}PbI_3$ Films Using Dynamic Spin-Coating. *Materials* **2021**, *14*, 6422. https://doi.org/10.3390/ma14216422

Academic Editors: Ana Pilar Valerga Puerta, Severo Raul Fernandez-Vidal, Zhao Zhang and Umberto Prisco

Received: 1 October 2021
Accepted: 25 October 2021
Published: 26 October 2021

Publisher's Note: MDPI stays neutral with regard to jurisdictional claims in published maps and institutional affiliations.

Copyright: © 2021 by the authors. Submitted for possible open access publication under the terms and conditions of the Creative Commons Attribution (CC BY) license (https://creativecommons.org/licenses/by/4.0/).

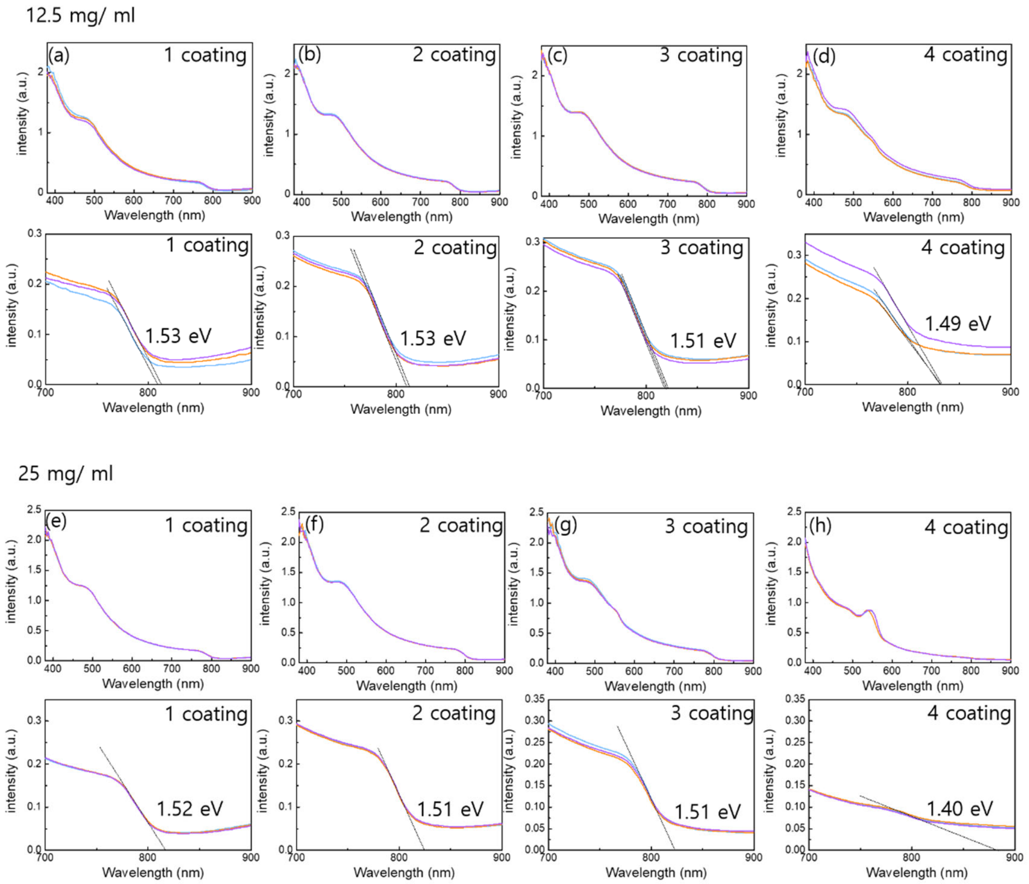

Figure S1. The calculation of bandgap of FAMAPbI₃ of FAI=12.5 with (**a**) 1 coating, (**b**) 2 coating, (**c**) 3 coating, (**d**) 4 coating and 25 mg/ml with (**e**) 1 coating, (**f**) 2 coating, (**g**) 3 coating, (**h**) 4 coating .

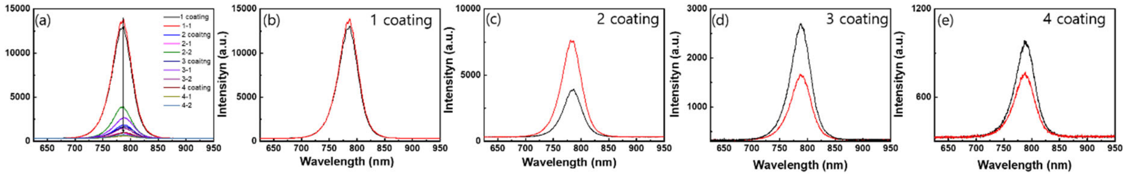

Figure S2. PL spectra of FAMAPbI₃ film of FAI=12.5mg/ml with different cycle of coatings (**a**) and PL spectra of FAMAPbI₃ film of FAI=12.5mg/ml with (**b**) 1 coating, (**c**) 2 coating, (**d**) 3 coating, (**e**) 4 coating.
